# Temporal dynamics of SARS-CoV-2 phylogenetic diversity in Central Brazil reveals evolutionary shifts among variants of concern during the pandemic

**DOI:** 10.3389/fmicb.2025.1639187

**Published:** 2025-08-13

**Authors:** José Alexandre Felizola Diniz-Filho, Rhewter Nunes, Cintia Pelegrineti Targueta, Ramilla dos Santos Braga Ferreira, Amanda Melo-Ximenes, Renata de Oliveira Dias, Juliana Santana de Curcio, Daniela Melo e Silva, Menira Borges de L. Dias e Souza, Fabíola Souza Fiaccadori, Elisângela de Paula Silveira Lacerda, Cristiana Maria Toscano, Thiago Fernando Rangel, Mariana Pires de Campos Telles

**Affiliations:** ^1^Departamento de Ecologia, Instituto de Ciências Biológicas, Goiânia, Brazil; ^2^Universidade Estadual de Goiás, Unidade Universitária de Iporá, Iporá, Brazil; ^3^Departamento de Genética, Instituto de Ciências Biológicas, Universidade Federal de Goiás, Goiânia, Brazil; ^4^Hospital Veterinário, Escola de Veterinária e Zootecnia, Universidade Federal de Goiás, Goiânia, Brazil; ^5^Instituto de Ciências Exatas e Naturais, Universidade Federal de Rondonópolis, Rondonopolis, Brazil; ^6^Programa de Pós-Graduação em Genética and Biologia Molecular, Instituto de Ciências Biológicas, Universidade de Federal de Goiás, Goiânia, Brazil; ^7^Departamento de Biociências e Tecnologia (Microbiologia), Instituto de Patologia Tropical e Saúde Pública, Universidade Federal de Goiás, Goiás, Brazil; ^8^Departamento de Saúde Coletiva, Instituto de Patologia Tropical e Saúde Pública, Universidade Federal de Goiás, Goiânia, Brazil; ^9^Escola de Ciências Médicas e da Vida, Pontifícia Universidade Católica de Goiás, Goiânia, Brazil

**Keywords:** SARS-CoV2, phylogenetic comparative analyses, mean pairwise distances, phylogenetic eigenvectors, GISAID, VOCs

## Abstract

The COVID-19 pandemic has led to substantial health, economic, and social impacts worldwide, and now, after more than 5 years since the start of the pandemic, it is possible to retrospectively evaluate patterns of SARS-CoV-2 spread and its consequences. Here we investigate the temporal dynamics of SARS-CoV-2 phylogenetic diversity in Goiás State, Central Brazil, using genomic data from 8,937 viral sequences obtained from GISAID between March 2020 and October 2024. Phylogenetic diversity was assessed through median pairwise distances (MedPD) and phylogenetic eigenvector regression (PVR) derived from principal coordinate analysis (PCoA) of pairwise distances among sequences. Results show evolutionary shifts associated with the emergence of new variants of concern (VOCs), particularly Gamma and Omicron, corresponding to distinct peaks in phylogenetic diversity through time. The initial rise in MedPD coincided with the Gamma variant’s emergence in early 2021, while a more pronounced peak followed the spread of the Omicron variant in late 2021. Although a third peak appeared in late 2023, it was based on smaller sample sizes and did not correspond to a major VOC. Moreover, the temporal dynamics of MedPD tends to mirror the epidemiological characterization of the epidemic over time, including morbidity and mortality, reflecting the impact of vaccination in the disease burden of subsequent variants. The strong phylogenetic signal over time, reflected in the first PCoA axis, highlights the evolutionary trajectory of the virus. This study illustrates how genomic surveillance provides critical insights into viral diversification and public health responses during pandemics.

## 1 Introduction

The COVID-19 pandemic, associated with severe acute respiratory syndrome coronavirus 2 (SARS-CoV-2), has led to substantial health, economic, and social impacts worldwide (Castro et al., 2021; Ashmore and Sherwood, 2023; World Health Organization, 2023). During the initial phases of COVID-19 pandemic, in early 2020, significant emphasis was placed on the temporal dynamics of the disease expansion at different geo-political units (e.g., municipalities, states, or countries), especially in the context of estimating rates of disease progression and evaluating likelihood of severe disease leading to hospitalization and deaths, thus guiding health policy decisions (Verity et al., 2020; Almeida et al., 2023; Ferguson, 2020). In a second moment, understanding the geographical and temporal patterns of spread established the baseline to assess the impacts of non-pharmaceutical interventions, such as social distancing measures and mandatory mask use, as well as to support strategic planning and policy making of healthcare service provision (e.g., Britton et al., 2020) and, after 2021, once COVID-19 vaccines became available, of vaccination strategies (Muller et al., 2023; Ulrichs et al., 2024).

However, as expected, SARS-CoV-2 rapidly diversified into multiple lineages (Hill et al., 2022; González-Vázquez and Arenas, 2023), some of which were designated as “Variants of Concern” (VOCs) due to increased transmissibility, immune escape, or severity (e.g., Harvey et al., 2021; Tao et al., 2021; Zhao et al., 2022; Jung et al., 2022; Flores-Alanis et al., 2022; Hussain and Wu, 2024) The most relevant VOCs detected worldwide and in Brazil, which emerged in different countries as accumulated mutations spread freely within communities, were Alpha (B.1.1.7), Beta (B.1.351), Gamma (P.1), Delta (B.1.617.2), and Omicron (B.1.1.529) (Salehi-Vaziri et al., 2022; Carabelli et al., 2023). Identifying these mutation patterns and lineages requires significant investments in DNA sequencing and genomics surveillance and, in Brazil, the interest in such programs began in early 2020 (Candido et al., 2020; Xavier et al., 2020) but it mainly increased after the identification of P1 (Gamma) lineage in Manaus (Sabino et al., 2021; Faria et al., 2021; Souza et al., 2020; Perico et al., 2022), which coincided with a major outbreak peak nationwide in early 2021. This marked the second COVID-19 wave, with the highest increase in case numbers and severe events including hospitalizations and deaths due to higher infection rate (Coutinho et al., 2021). The virus continued to diversify, resulting in the evolution of new VOCs over time (Salehi-Vaziri et al., 2022). Despite the sharp decline in severe disease and deaths resulting from the start of the vaccination rollout, concerns about spike mutations and immune escape continued (Tao et al., 2021; Chaudhari et al., 2022; Chen et al., 2024; Souza et al., 2025).

More than 5 years after the start of the pandemic, it is possible to evaluate patterns of SARS-CoV-2 spread and its consequences. In the context of VOCs evolution, cumulative DNA sequencing data can be used to access patterns of evolutionary diversification and learn lessons that could inform more effective VOC identification and evolution mapping strategies, providing a basis for a more complex evaluation of the temporal and geographical components of lineage evolution (Shaddick et al., 2023). While tracking VOCs is essential and an effective way to evaluate epidemiological impact of viral evolution, other approaches that address diversification in a continuous temporal dimension could be helpful in avoiding pitfalls related to VOCs identification. Thus, our goal is to apply multiple approaches from phylogenetic comparative analyses to evaluate the temporal spread of new lineages of SARS-CoV-2 in Goiás State, Central Brazil, illustrating how the availability of continuous genomic data can provide more insights into viral diversification, enhance surveillance, and support public health responses and decision-making.

## 2 Materials and methods

### 2.1 Studied region

The state of Goiás covers about 340,000 km2 in Central/Midwest Brazil, with about 7 million people. A substantial portion of the population lives in Goiânia, the state capital (with a population of about 1.5 million), and in nearby municipalities (with a population of about 3 million in the metropolitan region). The remainder of its population is distributed among 246 municipalities, with populations ranging from fewer than 10,000 people to more than 360,000 people. Moreover, the country’s capital, Brasília, is located in an administrative region (the “Federal District”) within the state of Goiás, which increases the region’s total population to over than 10 million people and thus placing the state of Goiás among the main hubs for the early spread of SARS-CoV-2. Thus, the regional patterns of viral diversification over time are quite representative of patterns throughout the country and can be used to demonstrate comparative analyses of genomic data during the pandemic. The first cases of COVID-19 in the region were detected on February 26th, 2020 in Brasilia, and a few weeks later, the first cases were confirmed in Goiás State. The state government enforced strict social distancing measures when only a few imported cases were reported to the surveillance system. Slowly but surely, COVID-19 cases were confirmed throughout the state. By late May 2020, more than 4,000 cases had been recorded in Goiás, occurring in approximately 60% of its municipalities. An additional 10,000 + cases were confirmed in Brasília. In early 2023, when the pandemic was declared to be over by the World Health Organization (WHO), more than 25,000 deaths had been officially recorded in the state.

At the beginning of the pandemic, the State Agency for Research Support of Goiás (FAPEG, the “Fundação de Amparo à Pesquisa do Estado de Goiás”) issued an emergency call for projects to address the health crisis. One of our team members, MPCT received initial funding to support genomic analyses of SARS-CoV-2 even though the interest in variants of concern (VOCs) intensified following the discovery of the Gamma variant in Manaus in early 2021 (Faria et al., 2021; Sabino et al., 2021; Naveca et al., 2021). From that point onward, the State Secretary of Health of Goiás expanded financial support for genome surveillance efforts, enabling the sequencing of approximately 2,376 genomes using next-generation sequencing (NGS) platforms, including Illumina and Oxford Nanopore technologies. These sequences were subsequently deposited in the GISAID database and combined with additional publicly available sequences for further analysis (see below).

### 2.2 Data

On April 10, 2025, complete SARS-CoV-2 genome sequences and their associated metadata were downloaded from the GISAID database. Sequences from the state of Goiás, Brazil, were selected and filtered based on the following criteria: (i) complete genomes (defined by GISAID as sequences with >29,000 nucleotides and labeled as high coverage when containing <1% of undefined bases [Ns]); (ii) exclusion of low-coverage entries (>5% Ns); and (iii) inclusion of entries with complete collection dates only. A total of 8,937 complete SARS-CoV-2 genome sequences were aligned using MAFFT v7.503 (Katoh and Standley, 2013). The resulting alignment was then manually curated to identify misaligned regions or problematic sequences. Phylogenetic analysis was performed using the maximum likelihood (ML) method implemented in IQ-TREE 3 (Minh et al., 2020; Wong et al., 2025; see also Romano et al., 2025). Nucleotide substitution models were evaluated using ModelFinder (Kalyaanamoorthy et al., 2017), and the best-fit model (GTR + F + I + R7) was selected based on the consensus of AIC (Akaike Information Criterion), corrected AIC (cAIC), and BIC (Bayesian Information Criterion). Branch support values were obtained using 1,000 SH-aLRT and ultrafast bootstrap (UFBoot) replicates. The log-likelihood of the consensus tree was −283,306.674.

### 2.3 Analyses

**Figure 1:**
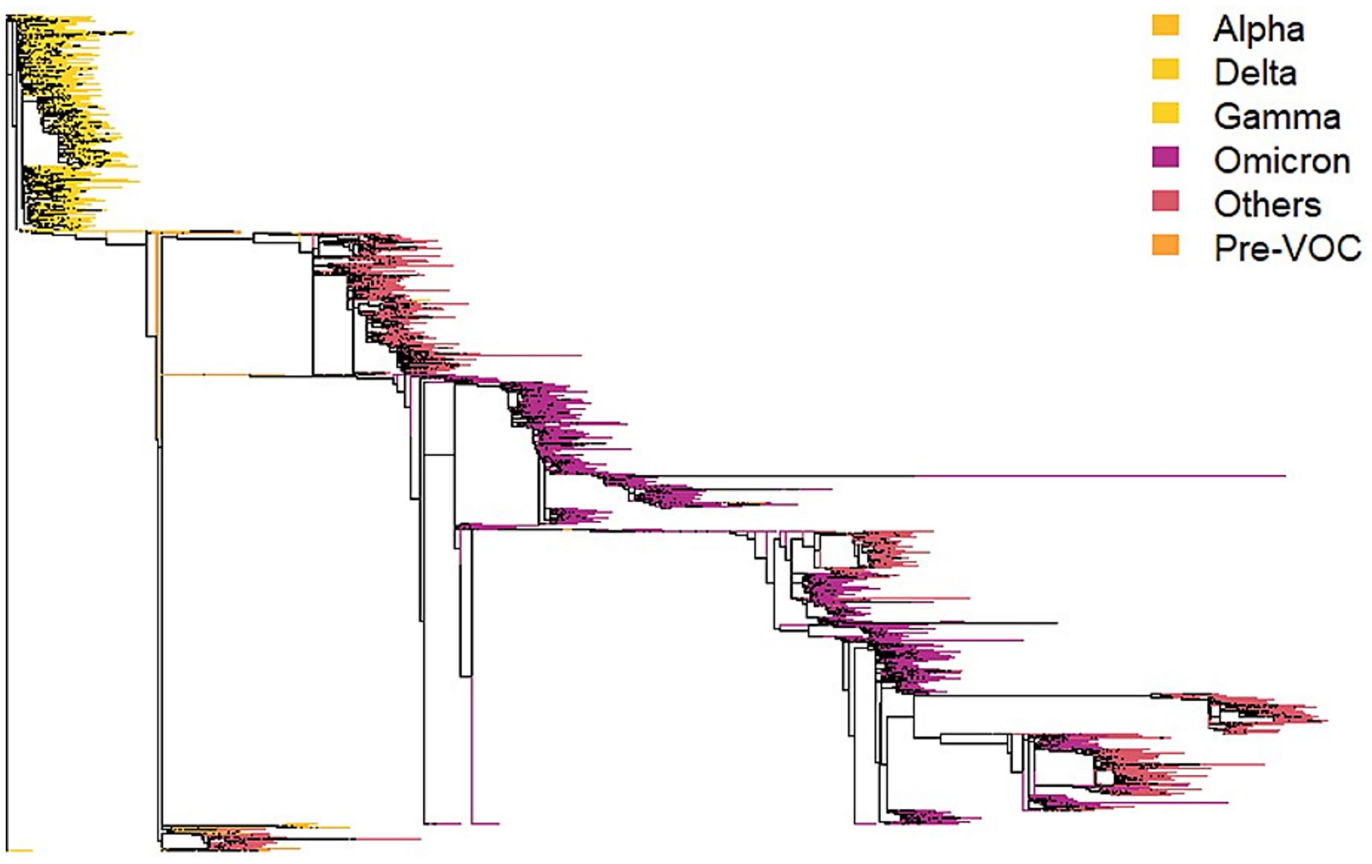
Maximum likelihood phylogeny for 8,937 sequences of SARS-COV2 from Goiás state, obtained in GISAID. The main categories of VOCs are shown as branch colors, coded as in the insert, along the scale of the first eigenvector extracted from the pairwise distances among sequences (see Figures 5, 6). Pre-VOC in SARS-CoV-2 refers to virus variants that emerged before the Variants of Concern (VOCs). The phylogeny clearly shows the transition from early Alpha, Delta and Gamma lineages (yellow tones) to later Omicron and related lineages, in purple. In our sample, classification of VOCs includes pre-Vocs (*n =* 105), Alpha (*n =* 40), Delta (*n =* 5), Gamma (*n =* 2,322), Omicron (*n =* 3,361) and others (3104). Pre-VOCs refers to Wuhan’s original type and first variants like B.1, B.1.1.28, B.1.1.33, whereas “others” include in general more recent variants after Omicron.

Pairwise patristic distances between the 8,937 sequences were obtained from the maximum likelihood phylogeny (Figure 1) and identified using the GISAID/Pango classification system.^1^ The tips were coded according to the main overall categories of VOCs (i.e., pre-VOC, Alpha, Gamma, Delta, Omicron, “others”) to facilitate the understanding of the temporal patterns of lineage diversification. In our sample, classification of VOCs includes pre-Vocs (*n =* 105), Alpha (*n =* 40), Delta (*n =* 5), Gamma (*n =* 2,322), Omicron (*n =* 3,361) and others (3104). Pre-VOCs refers to Wuhan’s original type and first variants like B.1, B.1.1.28, B.1.1.33, whereas “others” include in general more recent variants after Omicron.

The first goal of our study was to assess phylogenetic diversity over time. To achieve this, we grouped each sequence by month from March 2020 to October 2024. Due to the uneven sampling across months (ranging from 1 to 769 sequences, with a median of 85 sequences) and the skewed distribution of the distances within them, rather than using the more standard Mean Pairwise Distances (MPD) (Swenson, 2014; Tucker et al., 2017), we calculated the median distances and the 95% confidence intervals (MedPD). Even so, the correlation between MPD and median distances is high (r = 0.821), and thus the overall temporal patterns from the two metrics are qualitatively similar. For the 3 months without sampling, we performed simple interpolation by means between adjacent months, for graphical purposes only.

We also performed a Principal Coordinate Analysis (PCoA) on the pairwise distance matrix, extracting eigenvectors and eigenvalues to describe the main direction of evolutionary variation among sequences. This served as the basis for a Phylogenetic Eigenvector Regression (PVR; Diniz-Filho et al., 1998, 2012). We then related the first eigenvector to the sampling date (consecutive days from the first sampling), so that the R^2^ of this linear correlation estimates the phylogenetic signal of temporal shifts in SARS-CoV-2 diversification. To interpret the temporal patterns in MedPD and PVR, we used the PCoA to ordinate the mean phylogenetic distances among the main VOCs categories.

Finally, we also mapped the mean scores of the first eigenvector at the municipalities of Goiás state, for four different periods of about 1 year, from March 2020 to October 2024, to evaluate whether we could identify geographical patterns in the spread of VOCs along the pandemic. All phylogenetic comparative analyses were performed in several R packages (R Core Team, 2021) (see Supplementary material for details of analyses and packages).

## 3 Results

**Figure 2:**
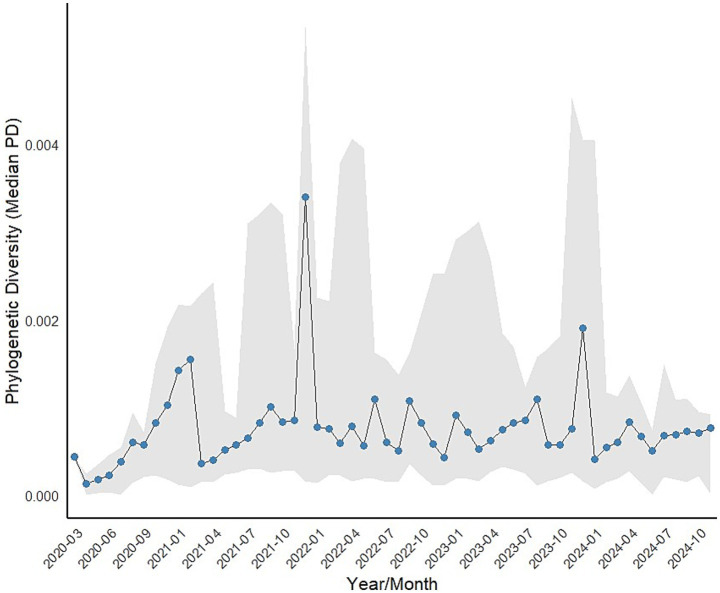
Temporal trend in median pairwise phylogenetic distances (MedPD) of SARS-COV-2 from March 2020 to October 2024, with gray shadow showing the 95% confidence interval around each MedPD.

As expected, the temporal curve of MedPD by month (Figure 2) reveals patterns that match the rise of new VOCs and their impact on the epidemiological characterization of COVID-19. Initially, we observed an increase in median phylogenetic distances up to a peak in January 2021, coinciding with the arrival of the Gamma VOC, which accounted for 60% of our samples. The distances then stabilize and oscillate, with the highest peak arising almost a year later, in November 2021, marked by the emergence of the Omicron VOC, which replaced Delta (with Omicron accounting for 49% of the sample, alongside Delta and other VOCs). There is a third peak in late 2023, but it is based on a relatively small sample size and does not contain any VOCs. Considering the period in which the samples were obtained, Gamma and Omicron are the predominant VOCs, with 26 and 38% of the samples, respectively.

**Figure 3:**
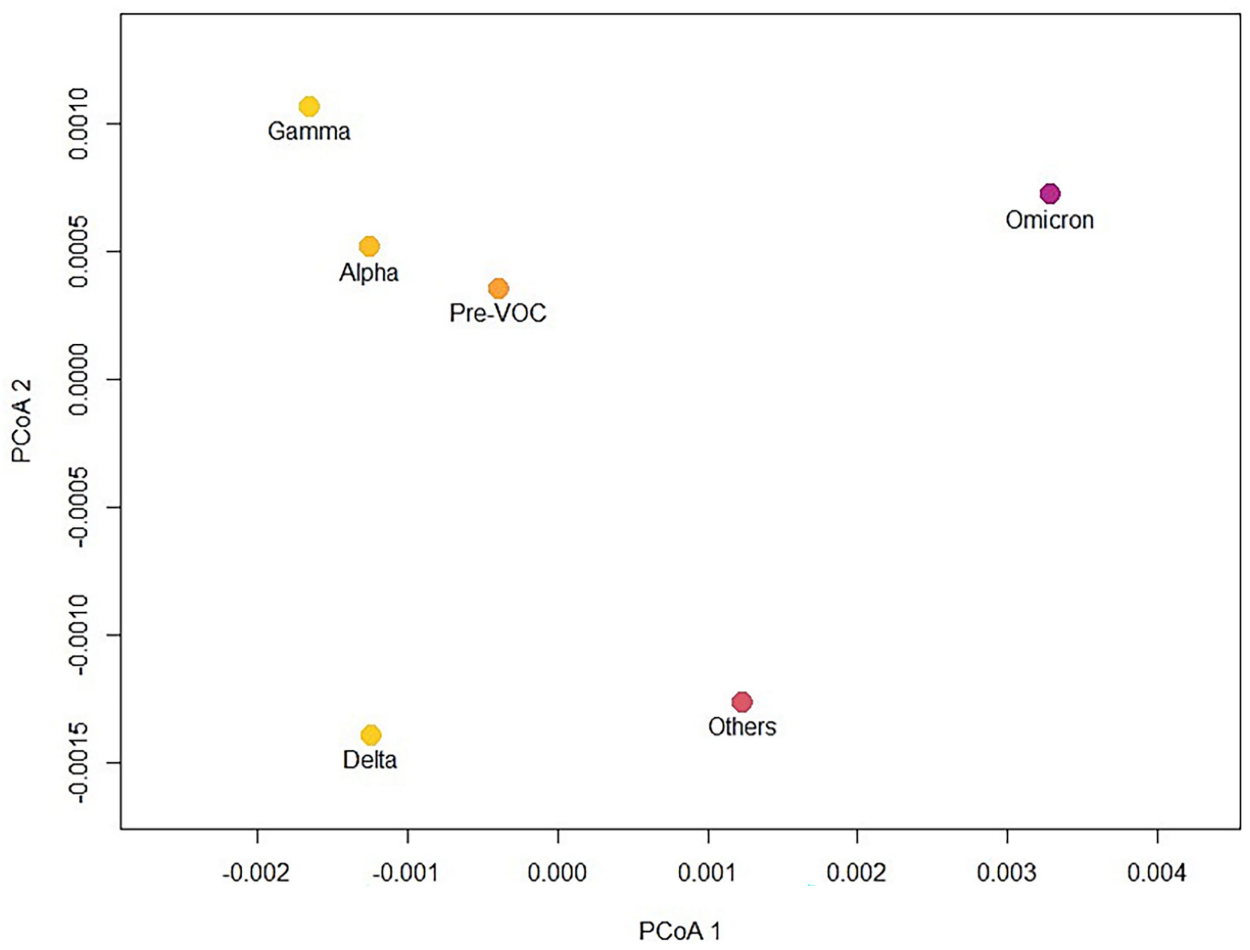
Principal coordinates analysis (PCoA) of mean pairwise phylogenetic distances among SARS-CoV-2 variants of concern (VOCs), highlighting the divergence of Omicron (explaining the peak in late 2021 shown in Figure 2) and the similarity of Alpha and Gamma which leads to a smaller peak of MedPD in early 2021.

The patterns in Figure 2 and the peaks in MedPD are understandable when considering the PCoA of the mean pairwise distances among VOCs (Figure 3), in which Omicron is indeed the most divergent lineage of SARS-CoV-2, which explains the highest peak in late 2021. On the other hand, Alpha and Gamma cluster closely, as observed in the phylogeny in Figure 1. Thus, although the arrival of Gamma represented a major threat and drove the largest peak in hospitalizations and deaths in early 2021, it does not appear as a high peak in phylogenetic diversity. Notice, however, that sequences identified as Omicron are also quite variable and have a larger variance along the first principal coordinate of phylogenetic distances (see Figure 5), which may also explain the higher peak in MedPD.

**Figure 4:**
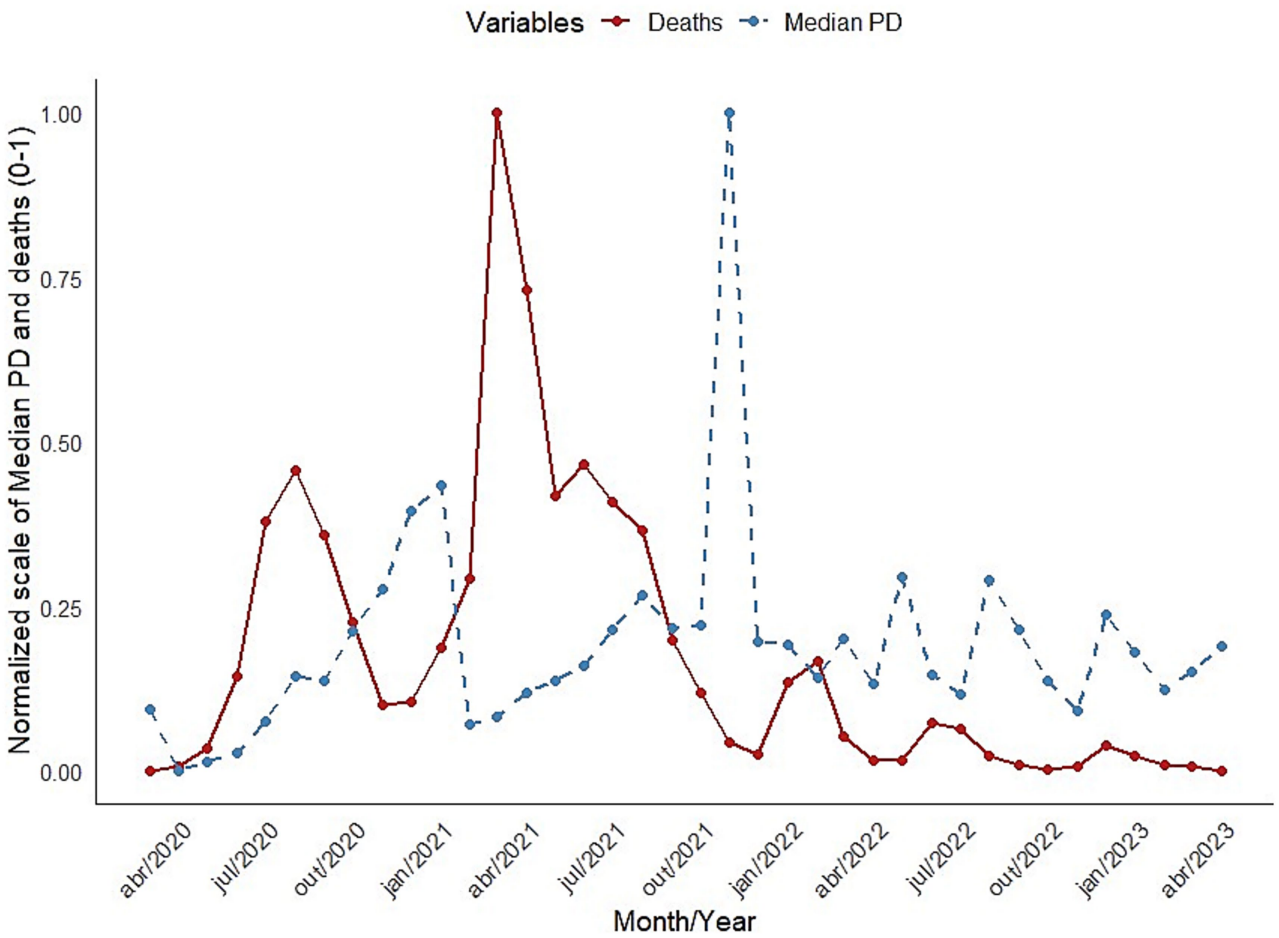
Temporal dynamics of MedPD (dashed blue line) and number of deaths (red line) in Goiás State, from March 2020 to April 2023, close to the end of the pandemic. The two variables were normalized to range between 0 and 1 for visualization purposes, but the death peak was equal to 4,175 deaths in March 2021.

It is also important to evaluate the relationship between MedPD and the epidemiological dynamics of COVID-19, especially the number of deaths. COVID-19 mortality data obtained from publicly available information systems of the Goiás State Health Department and MedPD over time are presented in Figure 4. While the relationship between the two series is not strictly linear or monotonic, and a simple correlation between the series does not yield any significant results, it may provide interesting insights for future surveillance programs regarding delayed peaks in the number of deaths and the indirect effects of vaccination.

**Figure 5:**
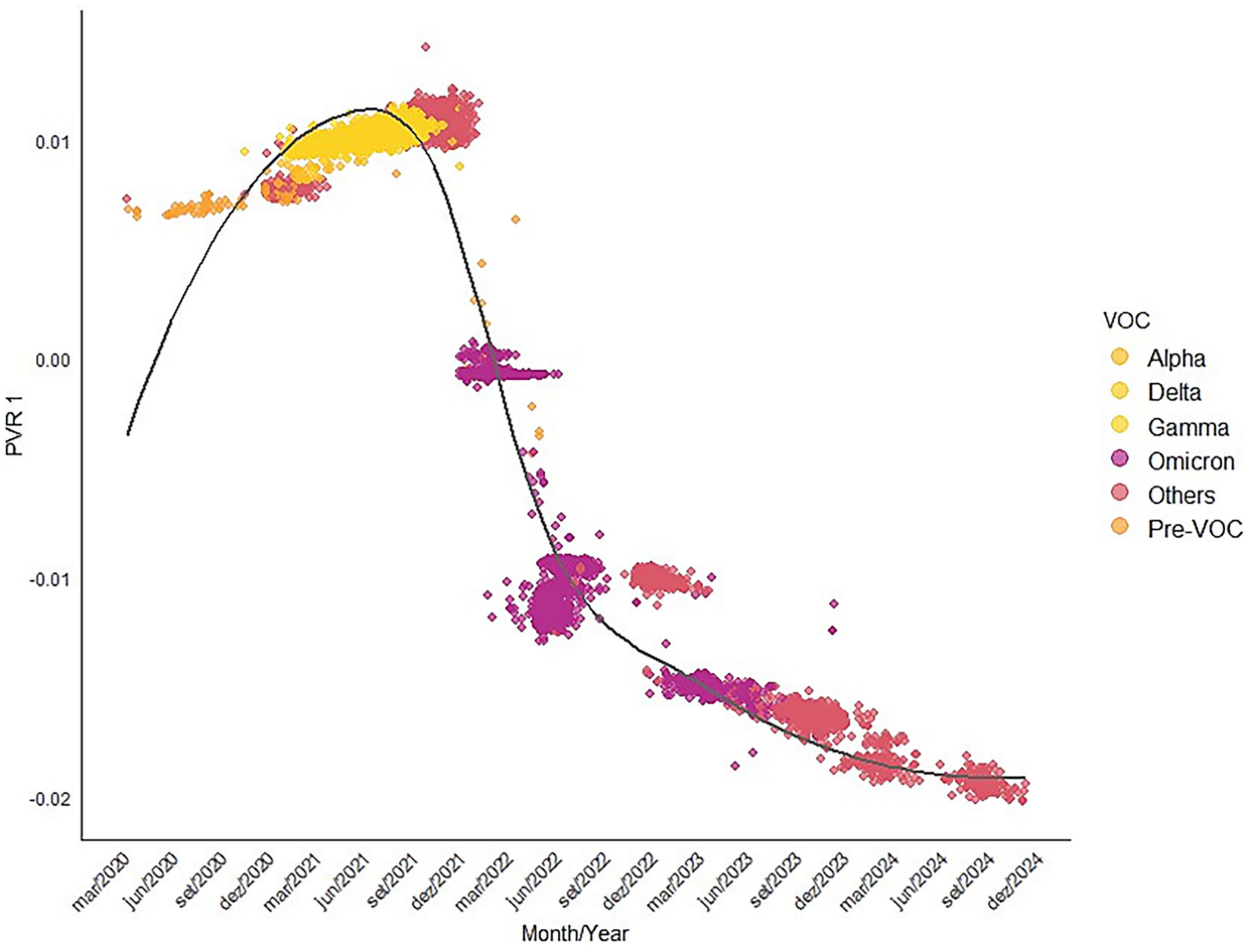
Temporal patterns in the first principal coordinate of the pairwise phylogenetic distances, mapping the VOCs of each sample through time. This first component explains 79.9% of the phylogenetic structure, and the correlation with days since the beginning of the pandemic was equal to 0.8.

The first eigenvector from the pairwise phylogenetic distances among the 8,937 sequences explains 79.9% of the distances variance, making it an adequate synthesis of the phylogenetic patterns in the data. Indeed, it is possible to observe the replacement of the VOCs over time along this axis (Figure 5). The phylogenetic signal in days since the start of the pandemic was equal to R^2^ = 0.64%.

**Figure 6:**
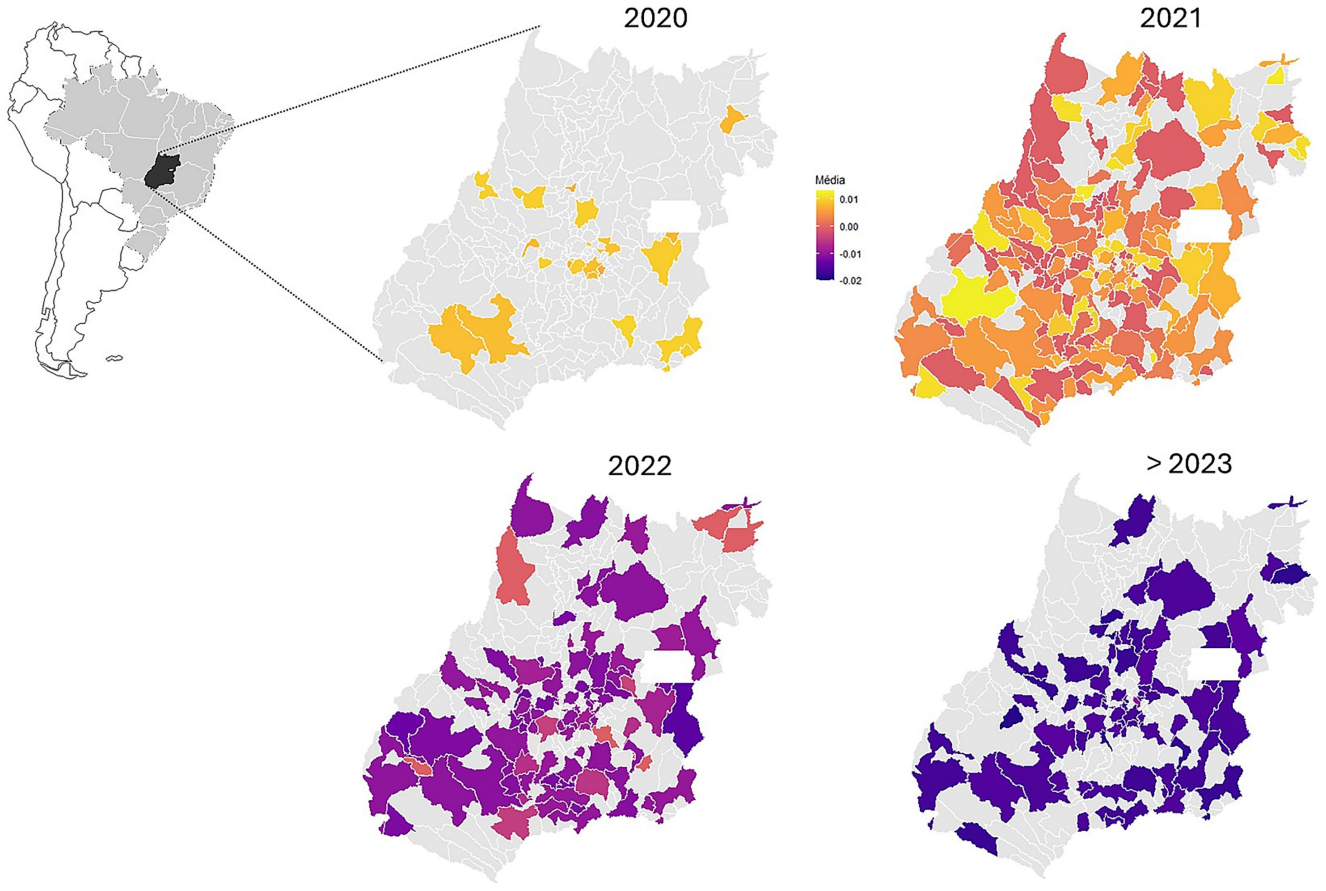
Geographic patterns in the first principal coordinate of the pairwise phylogenetic distances (see Figure 5) for distinct time periods throughout the pandemic. The sequence of maps reveals the geographic spread of the lineages along the main component of diversification, which ranges from the first Alpha and Gamma variants up to early 2021. These lineages appear on the yellow side of the color spectrum across the eigenvector and spread toward the more negative values, especially the Omicron variant in 2022, which appears in purple/blue.

Note also that the maps of the mean scores of the first principal coordinate of the pairwise phylogenetic distances show that in the sample used, during the first year of the pandemic (March 2020 up to February 2021) the Alpha, Gamma, and Delta lineages were sampled only in a few municipalities (Figure 6). However, in the second year, when DNA sequencing strategy increased, the Omicron group and its variants were already widespread in Goiás, and in the last 2 years of the pandemic, they were replaced by other VOCs at the more negative extreme of the first eigenvector. Figure 6 reveals that in 2021, the geographic distribution of SARS-CoV-2 lineages was predominant across all state of Goiás. However, the northern and northeastern regions exhibited a lower impact in terms of the geographic spread of the lineages in all years, which may be associated with lower population density in those areas.

## 4 Discussion

The COVID-19 pandemic presented several challenges for researchers, epidemiologists, and policymakers, as decisions had to be made despite the significant uncertainty regarding viral transmission patterns and their drivers. Moreover, when VOCs started to emerge, the situation became even more complex. The focus was on evaluating how the evolution of new lineages would allow immune escape and reinfection, and whether the available vaccines would remain effective (Carabelli et al., 2023; Bian et al., 2022). Now, more than five years after the start of the pandemic, with more consolidated data and a better understanding, it is possible to evaluate patterns and determine how alternative methodologies could be helpful for surveillance programs, particularly those with access to high-throughput sequencing technologies.

When considering VOCs, the idea has been to isolate them and check for adaptive shifts, mainly in the spike protein sequence, that could increase the transmission or infection rates of new lineages. Although classifying emerging lineages can be useful and help understand epidemiological curves of cases, hospitalizations, and deaths, it is an inadequate description of the diversification process in SARS-CoV-2. In fact, SARS-CoV-2 strains carrying point mutations in the spike gene emerged in the Brazilian state of Amazonas, likely due to viral spread from other countries. These mutations suggest the action of evolutionary pressures acting specifically on this region of the viral genome (Naveca et al., 2021).

Here, our focus is to shift from a more “discrete” view of this process, in which lineages are identified and monitored, to a more continuous approach, in which phylogenetic diversity is monitored using an established framework of phylogenetic comparative analyses and community phylogenetics (Tucker et al., 2017; Swenson, 2014). Ideally, these metrics will provide a more explicit pattern for genomic surveillance over time. Rather than monitoring the emergence of a new VOC, which depends on taxonomic issues, a more continuous phylogenetic diversity estimate will be used (measured by pairwise distances among samples within a given time period) to anticipate potential threats due to new VOCs.

The temporal patterns of MedPD in the state of Goiás reveal the usefulness of evaluating the diversification dynamics of SARS-CoV-2 and its VOCs, providing a simple approach to genomic surveillance, allowing it to discuss them in distinct ways. This study provides a retrospective overview of the distribution of SARS-CoV-2 variants in the 5 years after the end of the COVID-19 pandemic, highlighting the importance of accumulating genomic data for robust analytical approaches. Notably, this research group has contributed since the early stages of the pandemic, particularly through the work of Targueta et al. (2022), who reported the identification of the Delta variant and the optimization of the resequencing methodology. The study demonstrated the predominance of the Alpha and Gamma variants in the state of Goiás between November 2020 and July 2021, based on the real-time analysis of 318 samples.

In general, it is possible to observe from Figure 2 that there are cycles of MedPD, in which the increase in diversity leads and coincides with peaks in epidemiological events and stabilizes when a given VOC becomes dominant, i.e., when diversity is reduced. These peaks in MedPD are also evident when considering the divergence among VOC lineages. For instance, the PCoA analysis of the mean pairwise distances among VOCs (Figure 3) shows that Omicron is indeed the most divergent lineage, explaining the highest peak in late 2021. Some of the sequence variations in Omicron VOC are also related to the epidemiological characteristics of this variant, making it more transmissible (mutations increasing affinity of the ACE2 receptor) (Chatterjee et al., 2023; Planas et al., 2024), but were less lethal. It is important to note that the expansion of vaccination also reduced mortality during the increase in the number of Omicron cases. Indeed, the Omicron appears isolated in the bidimensional ordination space with the first two principal coordinates. The Omicron variant has the highest number of mutations particularly in the spike protein, compared to other VOCs (Parsons and Acharya, 2023). This represents a substantial increase in the number of genetic alterations compared to its predecessors, reflecting a high mutation rate, the emergence of multiple sublineages, immune evasion capacity and increased transmissibility (Hill et al., 2022). Pre-VOC lineages are centrally located in this space, reflecting their ancestral status, with Alpha and Gamma clustering closely. Thus, although the emergence of Gamma represented a major threat and drove the largest peak in hospitalization and death in early 2021, it did not generate a high peak due to its phylogenetic similarity with Alpha, which dominated the first epidemiological peak in mid-2020.

Another interesting aspect of the temporal patterns in MedPD is their relationship with epidemiological dynamics, especially with the number of COVID-19 deaths, as shown in the Figure 4. Due to the distinct factors involved in the time series of deaths, which create much more complex dynamics, a simple correlation between the series of MedPD and the monthly number of deaths, even with a time lag of 1 or 2 months, does not yield any significant results. However, a visual inspection of the series over time reveals that part of the failure of standard correlation analyses may be due to non-stationary processes underlying the patterns. Indeed, visually inspecting the two series provides useful information for further surveillance programs. For instance, the first peak in deaths in July 2020 does not appear to be related to a new VOC, despite the continuous increase in MedPD that lead to the first peak in January 2021. This peak was followed by another peak approximately 1 month later. The monthly death rate then decreased throughout 2021. However, the highest peak in MedPD due to the Omicron variant was not followed by another mortality peak because, by that time, COVID-19 vaccines had been made available, and mass vaccination campaigns had taken place, reaching in high coverage in Goiás and protection the population against severe disease and death. Note that even if an effective vaccination program breaks the correlation between the rise of new VOCs and epidemiological parameters, there seems to be a small peak in mortality again about 1 month after the Omicron peak. After that, mortality gradually decreased toward negligible figures at the population level in early 2023 (e.g., Li et al., 2024).

Another new approach proposed here is the PVR (Phylogenetic Eigenvector Regression; Diniz-Filho et al., 1998, 2012) of temporal changes in lineages. The overall idea of PVR is to extract eigenvectors from a phylogenetic distance matrix that can be used as predictors in a regression model. This allows one to describe phylogenetic patterns (signal) in the data and account for an inflated Type I error rate in models due to autocorrelation. In the case of SARS-CoV-2 lineages, for example, the first principal coordinate of the phylogenetic distances explains 79.9% of the phylogenetic structure in the data, thus, it is possible to represent most of the phylogenetic structure along a single axis. When this axis is correlated with the successive days on which sequences appear, its explanatory power is 64%. This indicates a high temporal signal in phylogenetic diversification and, indeed, it is possible to map how emerging VOCs with high prevalence, such as Gamma and Omicron, tend to appear gradually in time. From a more technical perspective within the PVR framework, it is interesting to note that the 64% explanatory power of such temporal patterns is lower than that of the axis itself. This indicates a departure from purely neutral lineage diversification (see Diniz-Filho et al., 2012, 2015). As shown in Figure 5, the relationship between the first axis and time is nonlinear. This departure may be due to nonstationarity issues and diversification constraints, in which lineages tend to evolve slower than expected by time since divergence. This is indeed expected if non-neutral, adaptive evolution occurs by creating constraints in diversification, for instance, when a given lineage increases its prevalence and, in some way, becomes dominant (which is, by definition, the case of VOCs). This may also be related to sampling bias, as there is a concentration of samples in periods when particular VOCs, particularly Omicron, were more common. Thus, further investigations with larger samples covering more homogeneously the entire period of the pandemic may shed light on how the PVR framework can be used to identify adaptive, non-neutral, evolution of variants that would have the potential to become VOCs.

Our study provides a detailed temporal analysis of the phylogenetic diversity of SARS-CoV-2 in Goiás, revealing how diversification matches key epidemiological milestones during the COVID-19 pandemic, showing peaks in median phylogenetic distances by month and phylogenetic eigenvector aligned with the emergence of major VOCs, particularly before widespread vaccination. Our findings highlight the critical importance of continuous genomic surveillance for understanding the diversification dynamics of SARS-CoV-2 and in guiding evidence-based public health strategies, particularly in the context of epidemic-prone diseases. Further studies would expand the reasoning developed here by evaluating the synchronicity of phylogenetic metrics at multiple spatial scales (among Brazilian States or countries worldwide), evaluating how phylobetadiversity (i.e., Graham and Fine, 2008) would track waves of distinct lineages in an explicit geographic context Although the extensive genomic dataset provides robust insights, sampling biases and variations in sequencing intensity over time should be considered when interpreting the results. Hopefully, the alternative proposed here can complement traditional epidemiological analyses based on classifying and monitoring VOCs in discrete groups, offering critical tools for monitoring ongoing and future viral epidemic threats. Future studies integrating genomic, clinical, and vaccination data could further clarify how to apply these methods, especially the PVR, to assess the complex dynamics between SARS-COV-2 evolution and COVID-19 severity load.

## Data Availability

The datasets presented in this study can be found in GISAID Platform, and R scripts used for phylogenetic comparative analyses can be found in the Supplementary material.

## Footnotes

^1^ https://cov-lineages.org/resources/pangolin.html
