# Supplementary material for: Temporal dynamics of SARS-CoV-2 phylogenetic diversity in Central Brazil reveals evolutionary shifts among variants of concern during the pandemic
Source: Front Microbiol. 2025 Aug 13;16:1639187. doi: 10.3389/fmicb.2025.1639187 (PMC12380668; doi:10.3389/fmicb.2025.1639187)
Supplement: Supplementary file 1 [file Data_Sheet_1.pdf]

Supplementary Material

Diniz-Filho et al., Temporal Dynamics of SARS-CoV-2 Phylogenetic Diversity in Central Brazil Reveals Evolutionary Shifts Among Variants of Concern during the pandemic. *Frontiers in Microbiology*.

Genomic Data

The bioinformatics pipeline used for obtaining the phylogenetic tree involved retrieving genomic data available from public database, performing multiple sequence alignment, curating the alignment, and reconstructing the phylogeny, respectively. On April 10, 2025, complete SARS-CoV-2 genome sequences and their associated metadata were downloaded from the GISAID database (https://www.gisaid.org). Sequences from the state of Goiás, Brazil, were selected and subjected to stringent quality filtering using the following criteria:

(i) completeness, defined by GISAID standards as genome sequences containing more than 29,000 nucleotides and marked as high coverage when presenting fewer than 1% undefined bases (Ns);

(ii) exclusion of low-coverage entries (>5% Ns);

(iii) inclusion of entries that provided precise and complete collection dates (day/month/year). After applying these filters, a total of 8,937 high-quality SARS-CoV-2 genome sequences were retained for further analyses. Each of these genomes were automatically classified in GISAID in the lineages and VOCs based on Pango lineage classification system (https://cov-lineages.org/).

Phylogenetic Reconstruction

Sequences were aligned using MAFFT v7.503 (Katoh & Standley, 2013) with default parameters and the FFT-NS-2 algorithm. The resulting alignment was visually inspected and manually curated to resolve ambiguous positions, remove misaligned regions, and discard potentially problematic sequences with unusually high divergence or apparent sequencing errors. The curated alignment was then used as input for phylogenetic analysis. Phylogenetic analysis was performed using the maximum likelihood (ML) method implemented in IQ-TREE 3 (Minh et al., 2020; Wong et al., 2025). Nucleotide substitution models were evaluated using ModelFinder (Kalyaanamoorthy et al., 2017). The selection process involved testing 286 nucleotide substitution models, and the GTR+F+I+R7 substitution model was selected based on the consensus of AIC (Akaike Information Criterion), corrected AIC (cAIC), and BIC (Bayesian Information Criterion). Node support values were evaluated through 1,000 replicates each of the SH-like approximate likelihood ratio test (SH-aLRT) and ultrafast bootstrap approximation (UFBoot). Nodes with SH-aLRT ≥ 80% and UFBoot ≥ 95% were considered strongly supported. The log-likelihood of the consensus tree was –283,306.674. For the sake of

scientific reproducibility, the bash script used for this step of the pipeline is described below.

```
#!/bin/bash

# Declare variables
INPUT_FASTA="gisaid_hcov-19_2025_04_10_16_Goias.fasta"
ALN_FASTA="aln_sarscov2_go_2025_04_16.fasta"
MODEL="MFP"
BOOTSTRAP=1000
ALRT=1000
THREADS=100

# Multiple sequence alignment
mafft --auto "$INPUT_FASTA" > "$ALN_FASTA"

# Phylogenetic tree analyses
iqtree3 -s "$ALN_FASTA" -m "$MODEL" -bb "$BOOTSTRAP" -alrt "$ALRT" -nt
"$THREADS"
```

Median Phylogenetic Distances

Based on sample collect dates of each one of the 8937 SARS-COV2 sequences, we calculated the median phylogenetic distances for the lineages occurring in each month, starting in March 2020 (for a total of 56 *classified_months*). We started by building a presence-absence matrix of sequences by month (*PA* matrix, below), in which columns were the same sequences in the phylogeny (*tree_reordered*) and lines are months from 1 to 56. We then obtained the mean, median and CI95% for the pairwise phylogenetic distances (cophenetic function of *Ape* package; https://cran.r-project.org/web/packages/ape/index.html) obtained from the phylogenetic filtered (*keep.tip*) for those sequences found in each month.

```
MPD <-numeric()
varMPD <-numeric()
medPD <-numeric()
ic95L <-numeric()
ic95U <-numeric()

for (i in 1:(max(classified_months))){
  linha <- PA[i, ]
  sequences_presentes <- names(linha[which(linha==1)])

  if(length(sequences_presentes) > 1){
    tree_sub <- keep.tip(tree_reordered, sequences_presentes)
    pdist <-(as.dist(cophenetic(tree_sub),diag=F,up=F))

    MPD[i] <-mean(pdist) #mean pairwise distances
    varMPD[i] <-var(pdist)
```

```
    medPD[i] <-median(pdist) #median pairwise distances
    ic95L[i] <-quantile(pdist,p=0.025)
    ic95U[i] <-quantile(pdist,p=0.975)

  } else {

    MPD[i] == 0
    varMPD[i] == 0
    medPD[i] == 0
    ic95L[i] == 0
    ic95U[i] == 0
  }
}
```

The *medPD* and the 95% confidence intervals were then plotted (see Fig. 2 in the main text) and after normalization to range between 0 and 1 were paired with monthly deaths (see Fig. 4 in the main text).

Phylogenetic Eigenvector Regression

The Phylogenetic eigenVector Regression (PVR) (see Diniz-Filho et al. 1998, 2015) starts by obtaining a pairwise phylogenetic distance matrix from the phylogeny, using the cophenetic function of *Ape* package. The eigenanalyses of this matrix (i.e., a Principal Coordinate Analyses – PcoA) allows describing phylogenetic structure by a set of eigenvectors. In our analyses, due to large size of the pairwise distance matrix (8937 by 8937), the *Rspectra* package (https://cran.r-project.org/web/packages/RSpectra/index.html), using the following script.

```
#PVR
# PCoA optimized for large pairwise matrices

library(RSpectra)

dist_phylo <- cophenetic(tree_reordered)
D2 <- as.matrix(dist_phylo)^2
n <- nrow(D2)
H <- diag(n) - matrix(1, n, n) / n
B <- -0.5 * H %*% D2 %*% H
k <- 10
pcoa_rs <- eigs_sym(B, k = k)
eigenvalues_covid <- pcoa_rs$values
eigenvectors_covid <- pcoa_rs$vectors
```

The first axis of the PVR (*eigenvectors_covid[,1]*) is the main direction of variation in the phylogenetic, and in our analyses, it explains 79.9% of the variance in the phylogenetic distances (this is a measure of the "phylogenetic structure;

pcoa_rs$values[1] / sum(pcoa_rs$values)). We then mapped this first axis of the PVR through time (Fig. 5 in the main text) and a standard linear model (fitted the function *lm*) shows that the phylogenetic signal in days since the start of the pandemic was equal to R2 = 0.64%.

We created a continuous color scale based on this first axis of the PVR and used this scale to represent the evolutionary diversification of the lineages (see Fig. 1 in the main text) and recorded the respective color (in the same scale) for the mean value of the first axis of the PVR for each of the VOC, as classified by GISAID. In this color scale, VOCs appear on the more yellow side of the color spectrum across in early phases of the pandemic and spread toward the more negative values, especially the Omicron variant in 2022, which appears in purple/blue (see Fig. 5 in the main text). Finally, we also mapped in Goias municipalities the mean scores of each municipality in time (Fig. 6 in the main text) and assigned to each one the same color scale.

We used the same reasoning using multivariate ordination (PCoA) on the mean pairwise distances among VOCs classified in GISAID and plotted the first two axes to evaluate differentiation patterns among them (Fig. 3), helping interpreting lineage replacement creating peaks in MedPD
